# A Prospective Analysis of Vitamin D Levels in Pregnant Women Diagnosed with Gestational Hypertension after SARS-CoV-2 Infection

**DOI:** 10.3390/jpm13020317

**Published:** 2023-02-12

**Authors:** George Dahma, Marius Craina, Catalin Dumitru, Radu Neamtu, Zoran Laurentiu Popa, Adrian Gluhovschi, Cosmin Citu, Felix Bratosin, Vlad Bloanca, Satish Alambaram, Anthony Willie, Shiva Charana Kodimala, Rodica Anamaria Negrean, Elena Bernad

**Affiliations:** 1 Department of Obstetrics and Gynecology, “Victor Babes” University of Medicine and Pharmacy Timisoara, 300041 Timisoara, Romania; 2 Methodological and Infectious Diseases Research Center, Department of Infectious Diseases, “Victor Babes” University of Medicine and Pharmacy, 300041 Timisoara, Romania; 3 Department of Plastic Surgery, “Victor Babes” University of Medicine and Pharmacy Timisoara, Eftimie Murgu Square 2, 300041 Timisoara, Romania; 4 Bhaskar Medical College, Amdapur Road 156-162, Hyderabad 500075, India; 5 Igbinedion University, Faculty of General Medicine, Main Campus Mission Road 1090, Okada 302111, Nigeria; 6 MediCiti Institute of Medical Sciences, NTR University of Health Sciences, Hyderabad 501401, Telangana, India; 7 Faculty of Medicine and Pharmacy, University of Oradea, 410073 Oradea, Romania

**Keywords:** gestational hypertension, vitamin D, pregnancy infections, COVID-19, SARS-CoV-2

## Abstract

The great majority of existing studies suggests that the prognosis and outcomes of SARS-CoV-2 infections are improved with adequate vitamin D levels, with or without supplementation. Simultaneously, whether vitamin D supplementation during pregnancy lessens the chance of developing gestational hypertension is controversial. The objective of the present research was to evaluate whether vitamin D levels during pregnancy differ substantially among pregnant women who develop gestational hypertension following SARS-CoV-2 infection. The current research was designed as a prospective cohort following the pregnant women admitted to our clinic with COVID-19 until 36 weeks of gestation. Total vitamin D (25(OH)D) levels were measured in the three study groups in which pregnant women with COVID-19 during pregnancy and a diagnosis of hypertension after 20 weeks of gestation were considered the group of cases (GH-CoV). The second group (CoV) included those with COVID-19 and no hypertension, while the third group (GH) included those with hypertension and no COVID-19. It was observed that 64.4% of SARS-CoV-2 infections in the group of cases occurred during the first trimester, compared to 29.2% in the first trimester among the controls who did not develop GH. Normal vitamin D levels were measured at admission in a significantly higher proportion of pregnant women without GH (68.8% in the CoV group vs. 47.9% in the GH-CoV group and 45.8% in the GH group). At 36 weeks of gestation, the median values of 25(OH)D in the CoV group was 34.4 (26.9–39.7) ng/mL compared to 27.9 (16.2–32.4) ng/mL in the GH-CoV group and 29.5 ng/mL (18.4–33.2) in the GH group, while the blood pressure measurements remained over 140 mmHg among the groups who developed GH. There was a statistically significant negative association between serum 25(OH)D levels and systolic blood pressure (rho = −0.295; *p*-value = 0.031); however, the risk of developing GH was not significantly higher among pregnant women with COVID-19 if the vitamin D levels were insufficient (OR = 1.19; *p*-value = 0.092) or deficient (OR = 1.26; *p*-value = 0.057). Although insufficient or deficient vitamin D among pregnant women with COVID-19 was not an independent risk factor for the development of GH, it is likely that an association between first-trimester SARS-CoV-2 infection and low vitamin D plays a key role in developing gestational hypertension.

## 1. Introduction

It is typical for pregnant women to have low amounts of 25-hydroxyvitamin D circulating in their blood [1]. The active form of vitamin D has the ability to suppress renin biosynthesis and vascular smooth muscle cell proliferation and to regulate the transcription of genes that are linked to placental invasion, normal implantation, and angiogenesis [2]. Throughout pregnancy, the vitamin D physiology of the mother is changed, resulting in elevated levels of the vitamin D binding protein. Current theories suggest that the rise in 1,25(OH)2D is a biological response created to allow immunological tolerance via vitamin D pathways at the maternal–fetal interface, hence promoting healthy placentation [3].

Hypertensive disorders during pregnancy are a leading contributor to severe acute morbidity, long-term impairment, and death in both the mother and the developing baby [4]. It is believed that ten percent of pregnant women throughout the world suffer from hypertensive disorders, which presents a significant risk to the general population’s health [5]. Therefore, the presence or absence of vitamin D might play a probable role in the development of preeclampsia and prenatal hypertension [6]. However, a recent meta-analysis of clinical trials [7] that evaluated vitamin D supplementation during pregnancy found no strong evidence of a protective effect on gestational hypertension (GH). These trials were conducted to evaluate whether or not vitamin D supplementation during pregnancy reduces the risk of developing GH. Therefore, the hypothesis is yet to be demonstrated differently.

There is a lot of evidence to suggest that vitamin D may moderate immunological responses. Because COVID-19 exacts such a heavy toll on the immune system, there has been a lot of interest in the possibility that vitamin D might mitigate or avoid adverse immunological responses [8]. Vitamin D is capable of influencing several components of innate and adaptive immunity and might have the ability to affect the severity and consequences of COVID-19. Infections with SARS-CoV-2 lead to the downregulation of angiotensin-converting enzyme 2 (ACE2), which can result in a hazardous buildup of metabolites, leading to acute severe respiratory distress syndrome (ARDS), a feared complication of COVID-19 [9]. It has been discovered that vitamin D mitigates these interactions between SARS-CoV-2 and RAAS [10].

The vast majority of the available research indicates that SARS-CoV-2 infection prognosis and outcomes are better with sufficient concentrations of vitamin D, with or without supplementation [11,12]. However, some studies indicate that there are no significant differences based on vitamin D levels and/or that there are no improvements following supplementation [13,14]. Some people have even reported a lower rate of infection as a direct consequence of using the supplement in the past. In the context of the still ongoing COVID-19 pandemic, the current study aimed to determine whether vitamin D levels during pregnancy vary significantly among pregnant women who develop GH after SARS-CoV-2 infection.

## 2. Materials and Methods

### 2.1. Research Protocol and Ethics

The current research was designed as a prospective cohort to evaluate the levels of vitamin D after a pregnant woman infected with SARS-CoV-2 during the pregnancy period was diagnosed with GH. The study was multicentric, taking place at the Clinic of Obstetrics and Gynecology “Bega” from the “Pius Brinzeu” Emergency Clinical Hospital from Timisoara, affiliated with the University of Medicine and Pharmacy “Victor Babes” from Timisoara, Romania. The second study center was the Clinic of Obstetrics and Gynecology from the Emergency Clinical Hospital of Arad, Romania. During the COVID-19 pandemic, the “Bega” clinic was transformed into a COVID-19-specialized unit for pregnant women, receiving the majority of cases from the Timis county, with approximately 700,000 inhabitants. The second clinic functioned for both routine emergency and non-emergent cases, as well as having a separate unit for COVID-19 patients. The study was approved by the Ethics Committee of the “Pius Brinzeu” Emergency Clinical Hospital from Timisoara.

The Local Commission of Ethics for Scientific Research at the Timis County Emergency Clinical Hospital “Pius Brinzeu” in Timisoara, Romania, acts in compliance with European Union GCP Directives published 2005/28/EC and the rules of article 167 of Law no. 95/2006. The International Conference of Harmonisation of Technical Requirements for Registration of Pharmaceuticals for Human Use (ICH) and the recommendations guiding medical doctors in biomedical and clinical research involving human subjects from the Declaration of Helsinki act as guides.

The research protocol was to follow the pregnant women admitted to our clinic with COVID-19 until 36 weeks of gestation in order to monitor changes in blood pressure above the normal threshold during pregnancy. GH, as described by the American College of Obstetrics and Gynecology, was defined as a measured blood pressure over 140 mm Hg (systolic) or a diastolic pressure over 90 mm Hg after 20 weeks of gestation, with no prior history of high blood pressure [15]. After the diagnosis of GH was established, the patients who were accepted to participate in the study were measured for vitamin D levels twice (at the moment of GH diagnosis and before the moment of delivery). The blood tests were drawn and analyzed in the private sector without any affiliations, partnerships, or funding received to support this study. Total vitamin D (25(OH)D) levels in maternal serum were measured using an immunochemical technique with electrochemiluminescence detection, based on existing protocols [16]. A level between 29 and 20 ng/mL was deemed insufficient in vitamin D, while levels below 20 ng/mL were used to diagnose vitamin D deficiency during pregnancy [17].

The inclusion criteria accounted for patients’ age over 18 years old, a confirmed COVID-19 diagnosis by a positive PCR test [18], the onset of GH before 36 weeks of gestation, and the patients’ signed approval to participate in the study. Patients who lacked consent were not considered for inclusion, as well as underage patients or those who refused to sign the agreement. Other exclusion criteria comprised: (1) a prior diagnosis of GH or preeclampsia; (2) a history of essential hypertension; (3) SARS-CoV-2 infection after the onset of GH; (4) SARS-CoV-2 infection during the third trimester; (5) a diagnosis of severe GH where the blood pressure exceeds 160/110 mmHg; and (6) patients under medication that can affect the blood pressure or vitamin D levels. Only patients with mild and moderate COVID-19 severity were considered for inclusion. According to existing guidelines, mild COVID-19 was defined as a confirmed SARS-CoV-2 infection by real-time polymerase chain reaction test (RT-PCR) associated with “mild symptoms without dyspnea”. Moderate COVID-19 was defined as a positive RT-PCR test for SARS-CoV-2 associated with “clinical and radiographic evidence of lower respiratory tract infection with oxygen saturations that exceed 94%” [19].

### 2.2. Study Variables and Patient Groups

The study variables that were collected for analysis comprised general and obstetrical information (age, body mass index, number of pregnancies, number of births, history of pregnancy-associated conditions, number of existing comorbidities, smoking status, vaccination status, trimester of SARS-CoV-2 infection), data about nutritional supplements during pregnancy (vitamin D, calcium/magnesium, folate, iron, probiotics, dose of vitamin D taken during pregnancy, and duration of vitamin D supplementation), and measurements taken at diagnosis of GH and at birth (vitamin D levels and blood pressure levels). The information about vitamin D supplementation that was not found in the patients’ records was collected through a short survey.

The first group of cases included pregnant women who developed hypertension after 20 weeks of pregnancy, after testing positive for SARS-CoV-2 during the current pregnancy. The second group comprised controls of pregnant women without GH who were diagnosed with COVID-19 during the current pregnancy. Lastly, the third group was also included as a comparison group, comprising pregnant women without a history of COVID-19 during the current pregnancy but who developed GH. Patients were case-matched with a 1:1:1 ratio of cases with GH versus no GH, using the proportion of comorbidities and smoking status as matching criteria. The three study groups were defined as follows: (1) the group of patients with hypertension and COVID-19 was coded as “GH-CoV”; (2) the group of patients with COVID-19 and no hypertension was coded as “CoV”; and (3) the group of patients with hypertension and no COVID-19 was coded as “GH”.

### 2.3. Statistical Analysis

Data were organized using MS EXCEL and analyzed with IBM SPSS v.27 [20]. Prior to performing inferential statistics, a Kolmogorov–Smirnov analysis was used to determine the normal (Gaussian) distribution of continuous variables. The comparison was made with the Student’s t-test or ANOVA using mean and standard deviation values. Non-normally distributed data were represented as the median and interquartile range (IQR), and compared with the non-parametric Mann–Whitney U test or the Kruskal–Wallis test using a 0.05 threshold for statistical significance. The proportion between groups was represented as a number (n) and percentage (%), being compared with the Chi-square test or Fisher’s exact test if the assumption of the expected value was violated. Pearson’s and Spearman’s correlation coefficients were calculated between the study variables with significant differences between groups, and the odds ratio for developing GH in association with decreased serum 25(OH)D levels and SARS-CoV-2 infection was also calculated. The relationship between COVID-19 and the serum 25(OH)D levels in pregnant women with and without GH was analyzed, including 96 samples from the GH-CoV group and the CoV group. The analysis of systolic and diastolic blood pressure was stratified by each of the three study groups, including a total of 144 samples.

## 3. Results

### Background Analysis

At the end of the study period, a total of 144 pregnant women were eligible for inclusion in the patients’ analysis. The most prevalent comorbidity was anemia in more than 30% of all patients, followed by peripartum infections and gestational diabetes mellitus. It was observed that COVID-19 pregnant women who did not develop gestational hypertension had an overall significantly lower count of previous pregnancy-related complications (37.5% vs. 22.9% in the GH-CoV group and 25.0% in the GH group), as seen in Table 1.

Since case-matching was performed based on the number of comorbidities and smoking status, there were no significant differences between the study groups, where approximately 70% of all participants did not have comorbidities at all. Regarding the COVID-19 vaccination status, there were only 12 (25.0%) vaccinated patients in the GH-CoV group, 37.5% in the CoV group, and 29.2% in the GH group, with no significant differences. Approximately 75% of all studied patients who received COVID-19 vaccines got the Pfizer BNT162b2. It was observed, however, that among pregnant patients who developed GH after COVID-19, they were infected much earlier during pregnancy compared with the CoV group, with a median week of infection of 9.4 weeks of gestation compared to 14.8 weeks (*p*-value < 0.001). Similarly, 64.4% of SARS-CoV-2 infections in the GH-CoV group occurred during the first trimester, compared to 29.2% in the first trimester among the controls who did not develop GH (*p*-value < 0.001).

Although the difference in vitamin D supplementation between the three groups was not statistically significant, the difference among the CoV and the GH-CoV groups was significant (33.3% vs. 47.9%, respectively; *p*-value = 0.038). A similar difference was observed in calcium and magnesium supplementation between the CoV group and GH group (31.3% vs. 18.8%, respectively; *p*-value = 0.046). Other statistically significant differences were observed in the proportion of vitamin D dosage among the pregnant women who took vitamin D during the current pregnancy. A higher proportion of patients from the CoV group consumed a vitamin D dose higher than 4000 UI compared to the other two groups who developed GH (56.5% vs. 37.5% vs. 16.7%; *p*-value = 0.038). Similarly, the duration of supplementation was longer in the group without GH, with 45.5% taking vitamin D for more than 24 weeks, compared to 7.7% in the GH-CoV group and 16.7% in the GH group (*p*-value = 0.034), as seen in Table 2.

**Figure 1:**
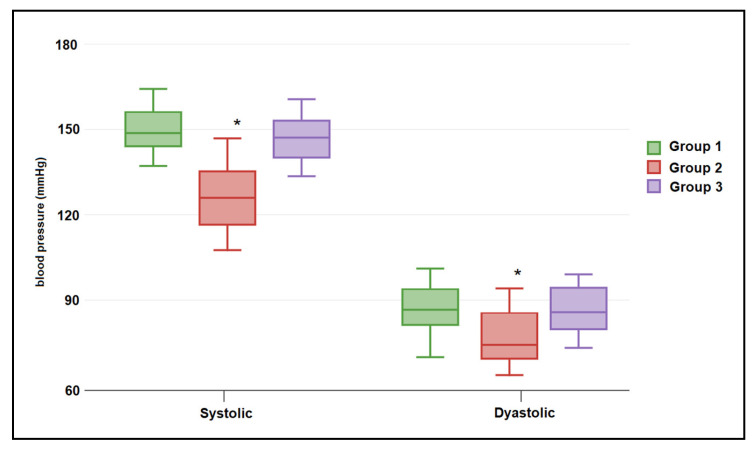
Systolic and diastolic blood pressure levels at initial measurement, stratified by study groups. Data presented as median (IQR) and analyzed using the Kruskal–Wallis test. * Dunn’s post-test analysis (significant at α < 0.05).

**Figure 2:**
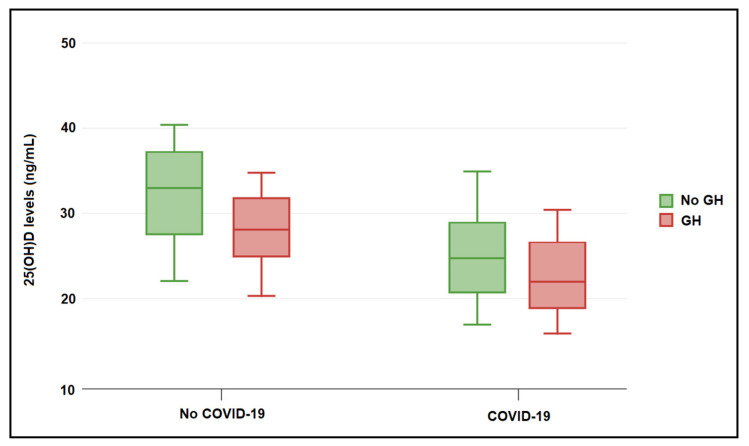
The relationship between COVID-19 and vitamin D levels in pregnant women with and without gestational hypertension (GH) at initial measurement. Data presented as median (IQR) and analyzed with the Mann–Whitney U test.

Table 3 describes the vitamin D measurement between the study groups. It was observed that vitamin D levels were significantly higher among pregnant patients from the CoV group who did not develop GH, with a median of 33.1 (28.1–38.7) ng/mL, compared to 22.4 (16.9–27.9) ng/mL in the GH-CoV group and 25.7 (19.4–29.6) ng/mL in the GH group (*p*-value <0.001), as seen in Figure 1. Normal vitamin D levels were measured in a significantly higher proportion of pregnant women without GH (68.8% vs. 47.9% in the GH-CoV group and 45.8% in the GH group; *p*-value = 0.044). The relationship between COVID-19 and the serum 25(OH)D levels in pregnant women with and without gestational hypertension is presented in Figure 2. Only the patients with COVID-19 (GH-CoV and CoV groups) were included in this analysis, with a total of 96 samples. A significant difference in median 25(OH)D values was observed based on COVID-19 status (27.9 (16.2–32.4) ng/mL in the no COVID-19 group vs. 34.4 (26.9–39.7) ng/mL in the COVID-19 group; *p*-value < 0.001), as seen in Figure 2. The median systolic blood pressure at initial measurement was 126.2 (108.0–136.3) mmHg in pregnant women without GH, 148.6 (141.3–156.8) mmHg in GH-CoV, and 145.9 (142.4–158.1) mmHg in GH. The same measurements were repeated at 36 weeks of gestation, observing a decrease in the patients with vitamin D deficit since vitamin D supplementation was started after the low initial measurements. Still, the median values of 25(OH)D levels were 34.4 (26.9–39.7) ng/mL in the CoV groups compared to 27.9 (16.2–32.4) ng/mL in the GH-CoV group and 29.5 (18.4–33.2) ng/mL in the GH group (*p*-value < 0.001), while the blood pressure measurements remained over 140 mmHg among the GH-CoV and GH groups.

The correlation analysis presented in Table 4 showed that vitamin D levels correlated significantly with the patient’s age, having a negative association (rho = −0.359; *p*-value = 0.022), as well as with the dose and duration of vitamin D supplementation. Remarkably, there was a statistically significant negative association between serum 25(OH)D levels and systolic blood pressure (rho = −0.295; *p*-value = 0.031). Other important findings were the significant correlation between the week of SARS-CoV-2 infection and the systolic blood pressure (rho = 0.172; *p*-value = 0.049), as well as the number of pregnancy-associated comorbidities and the patient’s age (rho = 0.301; *p*-value = 0.025).

**Figure 3:**
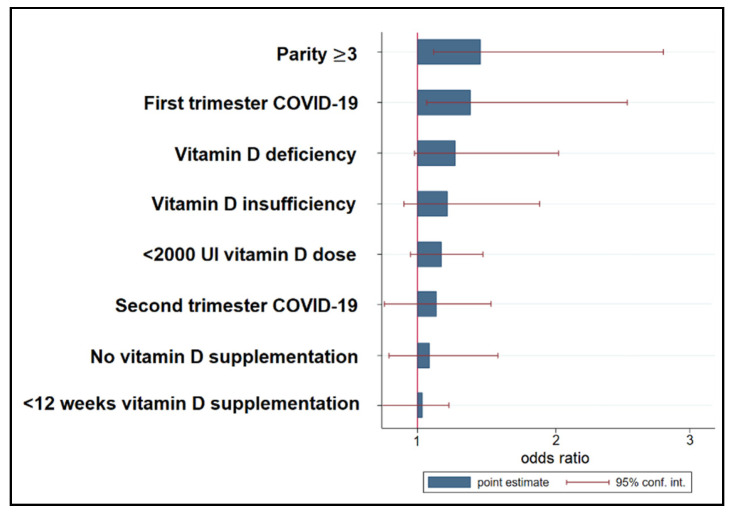
Risk factor analysis for gestational hypertension.

Although the correlation between vitamin D levels and systolic blood pressure was statistically significant, on regression analysis, the risk to develop GH was not significantly higher among pregnant women with COVID-19 if the vitamin D levels were insufficient (OR = 1.19; *p*-value = 0.092) or deficient (OR = 1.25; *p*-value = 0.057). Other statistically significant risk factors for developing GH during pregnancy were SARS-CoV-2 infection during the first trimester (OR = 1.37; *p*-value = 0.017) and carrying three or more pregnancies (OR = 1.51; *p*-value = 0.041), as seen in Table 5 and Figure 3.

## 4. Discussion

### 4.1. Important Findings and Perspectives

Several studies have shown that COVID-19 is more severe in pregnant women than in non-pregnant women, and there is a link between COVID-19 and gestational hypertension and preeclampsia, as well as a role of prenatal hypertension as a risk factor for infection with SARS-CoV-2 and associated consequences [21,22]. There were few instances of placenta infection and vertical transmission of SARS-CoV-2. Despite the fact that several studies have linked COVID-19 to placental inflammation and pregnancy problems such as gestational hypertension, other studies have shown no increase in COVID-19 severity among pregnant women [23,24]. Discrepancies across studies may be attributable to variations in demographic characteristics and prevalence of risk variables. The biggest question that arises is whether different populations such as those with dark skin and lower levels of vitamin D are less likely to benefit from the protective factor of vitamin D for the development of GH. What difference can vitamin D make among pregnant women after SARS-CoV-2 infection? Are they less likely to develop GH after COVID-19 if their vitamin D levels are high enough?

In both SARS-CoV-2 infection and the hypertensive diseases of pregnancy, including preeclampsia, the activity of ACE2 diminishes, leading to an imbalance between the levels of angiotensin [25]. COVID-19 may thus contribute to the pathophysiology of the hypertensive diseases of pregnancy, such as preeclampsia, through RAAS dysregulation, which is a primary mechanism of pregnancy hypertension. Additionally, hypertensive disorders during pregnancy are frequently connected with comorbidities, which may explain the severity of COVID-19 in these individuals. The immune system, inflammatory cytokines, and RAAS interact, and this interaction contributes to the development of hypertension. Patients with COVID-19 and preeclamptic patients exhibit elevated pro-inflammatory cytokines and a hyper-inflammatory state, which may be taken into account when assessing the illness severity in these patients [26]. Consideration must be given to the possibility that vitamin D’s immunological activities might offer anti-inflammatory protection independent of angiotensin 2 feedback modulation. It was not connected with SARS-CoV-2 infection, but the group with normal blood pressure had a larger percentage of pregnant women with normal concentrations of vitamin D, which inhibits gestational hypertension.

The initial hypothesis that we had was that appropriate vitamin D levels would reduce the rise in blood pressure that occurs during the third trimester of pregnancy. This was in line with the findings of a number of observational studies that suggested an association between vitamin D deficiency or low levels of active vitamin D and an increased risk of the hypertensive disorders that occur during pregnancy [27]. It has been hypothesized that because of the impact that vitamin D has on the renin–angiotensin system, it may help to maintain healthy blood pressure; however, research conducted on individuals who were not pregnant did not find any evidence to support the hypothesis that a low vitamin D level is linked to hypertension [28,29]. In addition, a recent population-based observational study conducted in Sweden demonstrated that 25(OH)D concentrations during the first trimester were positively associated with SBP (though not DBP) trajectory across pregnancy. This was the case despite the fact that higher levels of 25(OH)D were associated with a lower risk of preeclampsia and gestational hypertension. These data, which at first glance seem to be in conflict with one another, imply that the impact of vitamin D on the risk of preeclampsia may be mediated by alternative routes (such as immunomodulation) that are unrelated to blood pressure. Even in populations who are not pregnant, there is a lack of consistency in the data found in the scientific literature on the possible positive benefits of vitamin D supplementation on blood pressure and other health issues [30,31].

The increased risk of gestational hypertension seen in women with higher levels of 25(OH)D was somewhat unexpected in another study. Only one previous cohort research has investigated gestational hypertension apart from preeclampsia. Shand et al. [32] showed a decreased OR of 0.6 but a broad 95% confidence interval for gestational hypertension among women with 25(OH)D levels >37.5 nmol/L vs. lower levels. Their OR was in the opposite direction of ours, but their sample was significantly smaller; only 22 of 221 patients had gestational hypertension [32]. Still, there is no biological explanation in our study for the link between increased 25(OH)D and the gestational hypertension risk that we observed. It is probable that when women were diagnosed or in the process of being diagnosed with gestational hypertension, they increased their multivitamin consumption, resulting in greater levels of 25(OH)D. However, throughout the first and second trimesters, normotensive women raised their average vitamin D consumption more than hypertensive women [33]. In light of the association between greater 25(OH)D levels and gestational hypertension, it is premature to conclude that increasing vitamin D consumption during pregnancy would be beneficial. In addition, although gestational hypertension can develop at any time after 20 weeks of pregnancy, 80% of diagnoses are made after 36 weeks of pregnancy; however, reverse causality in physiology cannot be eliminated. For instance, if the placental expression of 1-alpha-hydroxylase increases in the presence of hypertension, 25(OH)D levels in hypertensive women may be greater [34,35].

The combination of vitamin D and calcium does not seem to provide any extra advantage. Calcium needs daily administration and a large dose, which may raise the cardiovascular risk of pregnant women [36]. In fact, the most current ESC, World Health Organization (WHO), and ACOG Guidelines propose prescribing calcium supplements for pre-gestational calcium shortage without mentioning vitamin D, despite the fact that vitamin D may be recommended for avoiding preeclampsia [37]. Indeed, vitamin D insufficiency is related to a substantial number of risk factors for endothelial dysfunction and impaired vascular health. Alternatively, enough vitamin D consumption may aid in the maintenance of calcium homeostasis—which is inversely associated with blood pressure levels or directly inhibits the growth of vascular smooth muscle cells. Moreover, vitamin D may be a potent endocrine inhibitor of renin production and may regulate the renin–angiotensin system, which is essential for blood pressure regulation. In addition, vitamin D may affect the production of adipokines associated with endothelial and vascular health [38]. Regarding blood pressure regulation, once GH is established, the question that arises is whether pregnant women should be pharmacologically treated or not. Considering that many blood pressure treatments are prohibited during pregnancy, some guidelines suggest that only when the blood pressure becomes severe (160/110 mmHg), pharmacological treatment should be established [39]. On the other hand, a recent major trial suggests that the right systolic blood pressure above which pregnant women should be pharmacologically treated is 140 mmHg [40].

Recommendations regarding vitamin D consumption during pregnancy continue to be the subject of debate. The US Institute of Medicine recommends that pregnant and breastfeeding women consume 600 IU (15 g) of vitamin D per day, while the UK National Health Service recommends that all adults (including pregnant women) consume approximately 400 IU (10 g) per day, and the Health Council of the Netherlands suggests that all pregnant women take 400 IU (10 g) of vitamin D per day [2,41]. In other countries, pregnant women are advised to consume one teaspoon of cod liver oil each day, which provides 400 IU (10 g) of vitamin D. According to the World Health Organization, there is inadequate evidence to suggest that vitamin D supplementation for pregnant women decreases bad pregnancy outcomes [42]. According to the findings, beginning between 12 and 16 weeks into a pregnancy to take a vitamin D supplement at a rate of 4000 international units per day is the most effective way to achieve vitamin D sufficiency. This is necessary in order to achieve an optimal nutritional and hormonal vitamin D status throughout the duration of the pregnancy [27,43]. Nevertheless, our study might suggest that optimizing vitamin D levels during pregnancy can help prevent the development of GH and COVID-19 infection.

### 4.2. Study Strengths and Limitations

One of the biggest limitations of the current study is the limited sample size, since the incidence of gestational hypertension is low, in addition to the selection of only pregnant women who were infected with SARS-CoV-2 during the current pregnancy. Therefore, the statistical power might be affected. The next step would be to perform large, well-designed studies of vitamin D supplementation. Specifically, it is essential to determine if the effect of vitamin D supplementation varies by ethnic group and baseline 25-hydroxyvitamin D levels. The majority of research has included only persons of European descent. Another limiting factor of the current study is that vitamin D supplementation was self-reported by patients in accordance with the recommendation received by their gynecologist, therefore data might not be entirely accurate due to response bias. More research is required to establish whether or not COVID-19 is more severe in pregnant women and whether or not it has a role in the development of pregnancy-related problems.

## 5. Conclusions

It was observed that vitamin D deficiency and vitamin D insufficiency among pregnant women with COVID-19 may be an independent risk factor for the development of gestational hypertension. However, it can be hypothesized that the association between first-trimester SARS-CoV-2 infection and low vitamin D levels can influence the development of placental-mediated complications. Further research is required to investigate this association, including a larger sample and a healthy control group. Another question that arises is whether SARS-CoV-2 infection increases the risk of gestational hypertension as a primary mechanism, or whether hypertension could also be a risk factor for developing severe COVID-19.

## Figures and Tables

**Table 1:** General and obstetrical information.

	Group 1 GH-CoV (*n* = 48)	Group 2 CoV (*n* = 48)	Group 3 GH *(n* = 48)	Significance
Age, years (mean ± SD)	30.6 ± 5.8	29.8 ± 5.5	31.0 ± 5.9	0.581
BMI, kg/m^2^ (mean ± SD)	24.4 ± 3.4	23.7 ± 3.9	24.9 ± 3.6	0.271
Gravidity (n,%)				0.875
1	3 (6.3%)	5 (10.4%)	3 (6.3%)	
2	37 (77.1%)	34 (70.8%)	38 (79.2%)	
≥3	8 (16.7%)	9 (18.8%)	7 (14.6%)	
Parity (n,%)				0.768
1	2 (4.2%)	3 (6.3%)	3 (6.3%)	
2	39 (81.3%)	37 (77.1%)	41 (85.4%)	
≥3	7 (14.6%)	8 (16.7%)	4 (8.3%)	
History of pregnancy-associated conditions (n,%)				
Gestational diabetes mellitus	5 (10.4%)	6 (12.5%)	8 (16.7%)	0.654
Abnormal presentation	2 (4.2%)	0 (0.0%)	3 (6.3%)	0.234
PROM	4 (8.3%)	2 (4.2%)	1 (2.1%)	0.349
Anemia	19 (39.6%)	15 (31.3%)	15 (31.3%)	0.609
Peripartum infection	4 (8.3%)	5 (10.4%)	6 (12.5%)	0.799
Other maternal infections	3 (6.3%)	2 (4.2%)	3 (6.3%)	0.876
None	11 (22.9%)	18 (37.5%)	12 (25.0%)	0.230
Comorbidities (n,%)				0.492
0	33 (68.8%)	36 (75.0%)	34 (70.8%)	
1	5 (10.4%)	8 (16.7%)	7 (14.6%)	
≥2	10 (20.8%)	4 (8.3%)	7 (14.6%)	
Smoking (n%)	8 (16.7%)	6 (12.5%)	10 (20.8%)	0.548
COVID-19 vaccination (n,%)	12 (25.0%)	18 (37.5%)	14 (29.2%)	0.399
Types of COVID-19 vaccines (n,%)	(*n* = 12)	(*n* = 18)	(*n* = 14)	0.077
BNT162b2	9 (75.0%)	13 (72.2%)	11 (78.6%)	
mRNA-1273	1 (8.3%)	5 (27.8%)	0 (0.0%)	
Ad26.COV2.S	2 (16.7%)	0 (0.0%)	3 (21.4%)	
SARS-CoV-2 infection				
Moment of infection, week (median, IQR)	9.4 (5.2–12.3)	14.8 (11.3–17.9)	–	<0.001
First trimester (n,%)	31 (64.6%)	14 (29.2%)	–	<0.001
Second trimester (n,%)	17 (35.4%)	34 (70.8%)	–	<0.001
COVID-19 severity (n,%)				0.294
Mild	31 (56.3%)	32 (66.7%)		
Moderate	17 (43.8%)	16 (33.3%)		

PROM—premature rupture of membranes; BNT162b2—Pfizer BioNTech; mRNA-1273—Moderna; Ad26.COV2.S—Astra Zeneca; gravidity—the number of times a woman has been pregnant; parity—the number of times a woman has given birth to a live neonate.

**Table 2:** Vitamin supplementation profile.

	Group 1 GH-CoV (*n* = 48)	Group 2 CoV (*n* = 48)	Group 3 GH *(n* = 48)	Significance
Supplements taken during pregnancy (n,%)				
Vitamin D	16 (33.3%)	23 (47.9%)	18 (37.5%)	0.322
Calcium/magnesium	12 (25.0%)	15 (31.3%)	9 (18.8%)	0.367
Folate	40 (83.3%)	42 (87.5%)	39 (81.3%)	0.937
Iron	19 (39.6%)	22 (45.8%)	21 (43.8%)	0.820
Probiotics	9 (18.8%)	9 (18.8%)	12 (25.0%)	0.684
Others	5 (10.4%)	3 (6.3%)	3 (6.3%)	0.674
None	4 (8.3%)	3 (6.3%)	6 (12.5%)	0.553
Dose of vitamin D supplementation (n,%)	(*n* = 16)	(*n* = 23)	(*n* = 18)	0.038
<2000 UI	9 (56.3%)	5 (21.7%)	11 (61.1%)	
2000–4000 UI	1 (6.3%)	5 (21.7%)	4 (22.2%)	
>4000 UI	6 (37.5%)	13 (56.5%)	3 (16.7%)	
Duration of vitamin D supplementation (n,%)	(*n* = 13)	(*n* = 22)	(*n* = 18)	0.034
<12 weeks	5 (38.5%)	4 (18.2%)	8 (44.4%)	
12–24 weeks	7 (53.8%)	7 (31.8%)	7 (38.9%)	
>24 weeks	1 (7.7%)	10 (45.5%)	3 (16.7%)	
Unknown	3 (18.8%)	1 (4.3%)	0 (0.0%)	0.082
Initial vitamin D measurement				
Week of gestation (median—IQR)	21.4 (17.9–23.1)	22.9 (19.7–24.7)	20.5 (18.1–22.8)	0.106

Dose and duration of vitamin D supplementation were measured until the 36th week of gestation.

**Table 3:** Comparison of vitamin D measurements and blood pressure between study groups.

	Group 1 GH-CoV (*n* = 48)	Group 2 CoV (*n* = 48)	Group 3 GH *(n* = 48)	Significance
Initial vitamin D measurement, weeks of gestation (mean ± SD)	28.5 ± 6.3	25.8 ± 5.4	27.3 ± 5.1	0.066
Initial blood pressure measurement, weeks of gestation (mean ± SD)	28.5 ± 6.3	20.3 ± 5.5	27.3 ± 5.1	<0.001
Vitamin D measurement				
25(OH)D levelsϑ(ng/mL), median (IQR)	22.4 (16.9–27.9)	33.1 (28.1–38.7)	25.7 (19.4–29.6)	<0.001
Normal vitamin D levels (≥30 ng/mL) (n,%)	23 (47.9%)	33 (68.8%)	22 (45.8%)	0.044
Low vitamin D (n,%)	(*n* = 25)	(*n* = 15)	(*n* = 26)	0.182
Vitamin D insufficiency (20–29 ng/mL)	11 (44.0%)	11 (73.3%)	13 (50.0%)	
Vitamin D deficiency (<20 ng/mL)	14 (56.0%)	4 (26.7%)	13 (50.0%)	
Blood pressure measurement				
Systolic blood pressure (median, IQR)	148.6 (141.3–156.8)	126.2 (108.0–136.3)	145.9 (142.4–158.1)	<0.001
Diastolic blood pressure (median, IQR)	88.3 (82.5–97.1)	80.4 (73.5–85.6)	87.5 (83.2–95.7)	<0.001
At 36 weeks				
Vitamin D measurement				
25(OH)D levelsϑ(ng/mL), median (IQR)	27.9 (16.2–32.4)	34.4 (26.9–39.7)	29.5 (18.4–33.2)	<0.001
Normal vitamin D levels (≥30 ng/mL) (n, %)	34 (70.8%)	40 (83.3%)	37 (77.1%)	0.345
Low vitamin D (n, %)	(*n* = 14)	(*n* = 18)	(*n* = 11)	0.040
Vitamin D insufficiency (20–29 ng/mL)	10 (71.4%)	17 (94.4%)	6 (54.5%)	
Vitamin D deficiency (<20 ng/mL)	4 (28.6%)	1 (5.6%)	5 (45.5%)	
Blood pressure measurement				
Systolic blood pressure (median, IQR)	145.1 (126.9–155.6)	127.6 (107.8–135.3)	143.7 (125.9–156.8)	<0.001
Diastolic blood pressure (median, IQR)	87.9 (83.5–97.7)	80.1 (74.6–86.4)	87.1 (85.8–97.2)	<0.001

SD—standard deviation; IQR—interquartile range; initial vitamin D levels in the CoV group were measured during the late second trimester.

**Table 4:** Correlation analysis of the studied variables.

		Age	BMI	Dose	Duration	Vit D Levels	Systolic P	Diastolic P	# of PAC	Week of Infection
**Age**	Rho	**1**	0.238	0.120	0.149	-0.359	0.361	0.235	0.301	0.106
	*p*-value	**-**	0.082	0.662	0.273	0.022	0.042	0.067	0.025	0.463
**BMI**	Rho	0.238	**1**	0.042	0.077	0.264	0.246	0.238	0.226	0.174
	*p*-value	0.082	**-**	0.678	0.599	0.053	0.044	0.053	0.077	0.377
**Dose**	Rho	0.120	0.042	**1**	0.220	0.465	−0.059	−0.145	0.085	0.084
	*p*-value	0.662	0.678	**-**	0.072	0.006	0.150	0.124	0.746	0.506
**Duration**	Rho	0.149	0.077	0.220	**1**	0.612	−0.156	−0.169	0.056	0.056
	*p*-value	0.273	0.599	0.072	**-**	0.000 **	0.078	0.346	0.418	0.228
**Vit D levels**	Rho	−0.359	0.264	0.465	0.612	**1**	−0.295	−0.099	0.105	0.075
	*p*-value	0.022 *	0.053	0.006 **	0.000 **	**-**	0.031	0.206	0.338	0.338
**Systolic P**	Rho	0.361	0.246	−0.059	−0.156	−0.295	**1**	0.611	0.226	0.172
	*p*-value	0.042 *	0.044 *	0.150	0.078	0.031	**-**	0.000	0.104	0.049
**Diastolic P**	Rho	0.235	0.238	−0.145	−0.169	−0.099	0.611	**1**	0.176	0.122
	*p*-value	0.067	0.053	0.124	0.346	0.206	0.000	**-**	0.192	0.095
**# of PAC**	Rho	0.301	0.226	0.085	0.056	0.105	0.226	0.176	**1**	0.064
	*p*-value	0.025 *	0.077	0.746	0.418	0.338	0.104	0.192	**-**	0.595
**Week of infection**	Rho	0.106	0.174	0.084	0.056	0.075	0.172	0.122	0.064	**1**
	*p*-value	0.463	0.377	0.506	0.228	0.338	0.049	0.095	0.595	**-**

** Correlation is significant at the 0.01 level (two-tailed); * correlation is significant at the 0.05 level (two-tailed); BMI—body mass index; dose—vitamin D dosage during pregnancy; duration—vitamin D supplementation duration during pregnancy; P—pressure (systolic/diastolic); PAC—pregnancy-associated conditions.

**Table 5:** Risk factor analysis for gestational hypertension.

Risk Factors	OR	95% CI	Significance
Parity ≥ 3	1.51	1.22–2.78	0.041
First trimester SARS-CoV-2 infection	1.37	1.10–2.52	0.017
Vitamin D deficiency (<20 ng/mL)	1.26	0.97–2.09	0.057
Vitamin D insufficiency (20–29 ng/mL)	1.19	0.93–1.86	0.092
<2000 UI vitamin D dose	1.15	0.96–1.54	0.217
Second trimester SARS-CoV-2 infection	1.08	0.83–1.61	0.199
No vitamin D supplementation	1.04	0.85–1.63	0.224
<12 weeks vitamin D supplementation	1.02	0.68–1.23	0.461

OR—odds ratio; CI—confidence interval.

## Data Availability

The data presented in this study are available on request from the corresponding author.
